# 

**Published:** 1921-06-25

**Authors:** 


### Retirement of a Medical Officer of Health.

Dr. F. Stevens, M.A., M.D., medical officer of health for the Borough of Camberwell, has been compelled to tender his resignation owing to lameness. The Council granted him leave of absence, and though his general health improved, the lameness did not, and the difficulty of getting about and the pain are considered permanent and incurable. Dr. Stevens will retire at the end of the present financial year in March next, when he will have completed twenty-nine and a-half years' service under this Authority. Until his retirement Dr. Howell Wood Barnes, the assistant medical officer of health, will perform his duties. It is proposed to retain the services of Dr. Stevens as honorary consultant medical officer of health to the borough.

### St. John's Hospital, Keighley.

THE Keighley Board of Guardians, believing that a good deal of the bad feeling resulted in consequence of the name, has decided to change the name of their infirmary to St. John's Hospital. The Vice-Chairman, Mr. W. Smith, contends that as people are admitted who are prepared to pay the full amount asked for, it is not a Poor-law hospital or infirmary. Steps are now being taken to make the hospital as comfortable as possible, and arrangements have now been made at the hospital for the reception of paying maternity patients.

### Young Men's Christian Association.

At the close of the war a difficult position arose with the organisation of the Y.M.C.A., as although there remained an enormous amount of work to do, the friends at home, thinking the war was over, largely ceased to contribute. The financial position at one time appears to have been very serious, but the War Office, recognising claims in respect of services rendered, paid the Association £590,000, as is shown by a summary of the accounts from August 1914 to May 1920. The total sum handled by the Association during the war amounted to just under £22,000,000. This includes, of course, the large costs of goods purchased for sale in the various huts and canteens, in respect of which there was a turnover of £17,000,000. The contributions from the general public amounted to £2,800,000.

### The Book Market.

THE receipt of the following is acknowledged :—

H. Frowde and Hodder & Stoughton: "Orthopædic Surgery of Injuries." By Sir Robert Jones, K.B.E., C.B., F.R.C.S. 2 vols. £4 4s. net per set.

Liverpool Council of Voluntary Aid, 14 Castle Street: "Liverpool Social Workers' Handbook, 1921." 2s. 6d.

Chapman & Hall: "Œdema and Nephritis." By Martin H. Fischer. 55s. net.

Constable & Co., Ltd.: "Radiographic Technique." By T. Thorne Baker, A.M.I.E.E. 15s. net.

DR. DAVID LLOYD ROBERTS, a well-known Manchester practitioner, has left £125,677. There are bequests to several charities, and the residue of the estate is given to University College at Bangor for general purposes.

### General Nursing Council for Scotland.

AT the meeting of this Council, held at 13 Melville Street, Edinburgh, on Wednesday, June 15, the Registrar reported that no further communication had been received from the Scottish Board of Health in regard to the negotiations with the Board as to the existing nurses on the Board's Fever Register, and the Registrar was instructed to communicate with the Board and endeavour to have a settlement of the question at issue expedited.

Colonel Mackintosh presented the report of the Syllabus Committee, and this was approved after some discussion. In the absence of Dr. Fraser, Colonel Mackintosh also submitted the report of the Education and Examination Committee, which had been engaged in preparing lists of approved hospitals for the training of existing nurses under the Rules.

A Registration Committee was appointed consisting of Captain C. B. Balfour, C.B., Chairman of the Council (*ex officio*); Miss Nora Milnes, Vice-Chairman of the Council (*ex officio*); Dr. A. K. Chalmers, Dr. H. E. Fraser, Colonel D. J. Mackintosh, C.B., M.V.O., Miss Kathleen L. Burleigh, Miss A. Gill, R.R.C., Miss Elizabeth T. Jones, Miss Florence A. Merchant, Miss Margaret R. Stewart, and Miss Margaret N. White.

### Passing Events and Topics.

### A Memorial to Hahnemann.

Hahnemann House, which, together with a gift of relics of the founder of homœopathy, has been presented to the London Homœopathic Hospital by Mr. M. Stuart and his brother Mr. O. Stuart, will eventually contain the Hahnemann Museum.

### The Alternative to Day Nurseries.

Speaking at the annual meeting of the National Society of Day Nurseries, held on June 8 at 5 Carlton House Terrace, Viscount Burnham said the tendency for women to find employment in industry probably was not good; but still it would go on, and the alternative to the nurseries was the utter neglect of the children until they became of school age. On the whole it was better that this problem should be dealt with by voluntary means and with the individual sympathy and help that the workers for the Society were able to bring to bear.

### Co-ordination in Manchester.

A North-Western Federation of Child Welfare, comprising the counties of Lancashire, Cheshire, Cumberland, and Westmorland, is in process of formation. Membership is open to any body approved by the Ministry of Health. Manchester and Salford is one of the latest cities to agree to be represented, and the movement is supported by the Manchester and Salford Council of Social Service, which body has also passed a resolution urging that all voluntary and official bodies in Manchester and Salford engaged in this work should join the Council of Social Service.

### Presentation to Medical Superintendent.

On the completion of forty years' service as medical superintendent of the Ladywell Sanatorium and Infectious Diseases Hospitals at Salford, Dr. John W. Mullen was presented by members of the staff with an armchair, a leather suit-case, and a hall barometer. To mark Dr. and Mrs. Mullen's silver wedding, which is also this year, Mrs. Mullen was presented with some silver plate, and Miss Doreen Mullen with a gold watch bracelet. In making the presentation, Councillor Williamson said that Dr. Mullen had won the love, admiration, and affection of every member of the Corporation, and the undying gratitude of thousands of men, women, and children in the borough. Dr. Mullen, who is a Dublin man, was apprenticed to the late Sir William Stokes, and after qualification was medical superintendent of the Dublin Smallpox Hospital.

### Child-Welfare Fete.

A great effort to raise money for the promotion of infant and child welfare is to be made on Saturday, July 16, when a Fête will be held in the Royal Hospital Gardens, Chelsea, under the auspices of the Central Council for Infant and Child Welfare. This body was brought into existence through the efforts of the Joint Council of the Order of St. John and the British Red Cross Society for the purpose of promoting the well-being of children from the ante-natal period up to the age of sixteen. Sir Napier Burnett, Director of the Special Peace Fund Appeal of the Joint Council, is actively interesting himself in the Fête. An earnest appeal is made for gifts of the usual kinds of saleable articles. Offers should be sent to Miss Wilson, Secretary of the Council, at 20 Berkeley Street, W.

### The "Sanitas" Company, Limited.

The forty-third annual general meeting of the "Sanitas" Co., Ltd., was held at the Cannon Street Hotel on June 15, under the presidency of the Chairman, Mr. C. T. Kingzett, F.I.C., F.C.S. Notwithstanding the general slump experienced during the year under review the trade has on the whole been satisfactory, sufficing for the payment of the full dividend of 9 per cent. on the preference shares of the company, and 5 per cent. on the ordinary shares. In view of the increase in business the company has acquired and equipped a model additional factory at Hayes, and will make use of it as soon as the revival of trade justifies the action.

### Gifts and Bequests.

Mr. E. Parsons, of Newport: £50 to Royal Gwent Hospital.

Mr. A. W. Emms, of Thurcaston, has bequeathed £1,000 to the Faire Hospital, Leicester.

£500 has been left to the Homœopathic Hospital at Tunbridge Wells by Mrs. E. A. Scott of that town.

The residue of the estate of the late Mr. William Middleton, estimated at about £20,000, has been left to Edinburgh Royal Infirmary.

Mr. Henry Rutson, of Northallerton, Yorks, has left £1,000 each to Ruston Hospital, Northallerton, and the York County Hospital.

Mrs. Amelia Levy, of Gloucester Terrace, W., has left £100 to the Samaritan Free Hospital for Women and £50 to the Paddington Green Hospital for Children.

Mr. William Foulkes, of Shrewsbury, has left £250 each in stock to the Shrewsbury Eye, Ear, and Throat Hospital, the Salop Royal Infirmary, and Baschurch Convalescent Home.

Mr. G. W. Williams, of Llanfrechfa, Mon., has left £1,000 to the Royal Gwent Hospital, Newport, to endow a bed in memory of his son, Major Edmund Styant Williams, who was killed in the late war.

Subject to a life interest and some legacies, Mr. T. N. Wells, of Hampstead, has left his property to be divided equally between St. Columba's Hospital, Hampstead, and the Orphan Working School, Haverstock Hill.

Colonel F. J. Ryder, C.M.G., of Leeds, left the residue of his estate, valued at over £25,000, equally between Leeds Infirmary, the London, Guy's, St. Thomas's, St. Bartholomew's, and Charing Cross Hospitals, and Edinburgh University.

The Cambrian Railways Co. have contributed 200 guineas to the Montgomery County Infirmary at Newtown in appreciation of the services rendered in connection with the lamentable accident on January 26, when sixteen people were killed and a large number injured.

Mr. Walter William Shillitoe, of Rugby, has left the residue of his estate to the Hospital of St. Cross, Rugby, requesting that a bronze head in relief may be made from his photograph and placed in a suitable position in the hospital, and that a small bronze replica of this head be placed on each of the Shillitoe beds.

### THE COLLEGE OF NURSING (Limited by Guarantee).

### NORTHUMBERLAND AND DURHAM CENTRE, NEWCASTLE-ON-TYNE.

(Secretary, Miss M. TOYNE, 17 Windsor Terrace, Jesmond.)

FRIDAY, JULY 8, 3 P.M.—Excursion to Finchale Abbey. The Secretary requests all those who wish to join to send in their names to her at 17 Windsor Terrace *not later* than Monday, June 27, otherwise seats in the motor charabanc cannot be booked.

### SHEFFIELD CENTRE.

(Hon. Secretary, Mrs. BARNES, 34 Wilkinson Street.)

A WELL-ATTENDED special general meeting was held on JUNE 7. The most important item on the agenda was the granting of a scholarship, value £60. The candidate must be a member of the Sheffield Centre; she can choose her own subject, but must sit for her qualifying examination at College headquarters before the end of the present College year.

A series of picnics was arranged, the first of which will be held on SATURDAY, JULY 2. Particulars may be had from Mrs. Watson, Acting Secretary, 426 Glossop Road, Sheffield.

It was decided to arrange some gatherings during the winter, with a view to raising the necessary funds for a Nurses' Club, a concert, a jumble sale, and the forming of a Dramatic Society being agreed to.

### ABERDEEN CENTRE.

(Hon. Secretary, Miss E. ENGLAND, 337 Clifton Road.)

A GENERAL MEETING of the Aberdeen Centre of the College of Nursing, Ltd., was held on Thursday, June 16, at 8 P.M., at the Cowdray Club, Miss Hill in the chair. Miss Sheriff-Macgregor addressed the meeting on the history of the College, and of its various activities, making special mention of the fact that individual effort is required to make the College an outstanding success.

### IRISH BOARD.

THE monthly meeting of the Irish Board was held at 54 Fitzwilliam Square, Dublin, on June 16, Sir John Moore presiding. Dr. Dunne was appointed joint trustee of the Irish Board in place of Dr. Tate, who is unable to act. A suggestion from the Belfast Centre, that the Board meetings might be held alternately in Dublin and Belfast, was deferred for consideration at the July meeting. On the invitation of the Council of the Nation's Tribute to Nurses, Miss Hill was appointed College of Nursing representative on that body in succession to Miss Matheson, resigned. A letter from the Irish Nurses' Union was read relative to the Public Health Services of Ireland and the necessity for the enlightenment of public opinion on this subject. Miss Chisholm reported having attended meetings convened by the Union in this connection. It was agreed that the College should co-ordinate with the Union as far as possible. It was reported that two Irish candidates had entered for the examination on June 18 in connection with the sister-tutor studentships offered by the College. The desirability of offering a special Irish studentship next year was strongly emphasised.

### MATRONS' AND SISTERS' APPOINTMENTS.

ALL SAINTS' HOSPITAL, FINCHLEY ROAD.—Miss Noel has been appointed theatre sister. She was trained at the Middlesex Hospital.

CHILDREN'S ORTHOPÆDIC HOSPITAL, STOKE-ON-TRENT.—Miss Blore has been appointed ward sister. She was trained at Ancoats Hospital, Manchester, and has held the post of sister at Mansfield and District Hospital, sister at Ancoats Hospital, and ward sister at the Ethel Hedley Children's Orthopædic Hospital, Windermere.

CITY OF PETERBOROUGH ISOLATION HOSPITAL.—Miss A. H. Green has been appointed matron. She was trained at the Central London District Infirmary, Hendon, and has been sister at the Walthamstow Isolation Hospital, cubicle sister at the City Isolation Hospital, Norwich, assistant matron of the Bromley and Beckenham Hospital, and sister and deputy matron of the Hove Borough Sanatorium.

FULWOOD WORKHOUSE INFIRMARY.—Miss Gwendoline E. Green has been appointed night superintendent-nurse. She was trained at Fulwood Workhouse Infirmary, and has been night sister and maternity sister at Southmead Infirmary, Bristol.

GUEST HOSPITAL, DUDLEY.—Miss A. Wolstenholme has been appointed theatre sister. She was trained at the Royal Infirmary, Sheffield, and the Jessop Hospital for Women.

INFECTIOUS DISEASES HOSPITAL, LITTLE BROMWICH.—Miss F. Browne Gray has been appointed night sister. She was trained at the Birmingham General Hospital, the Eastern Fever Hospital, and the National Hospital, Queen Square. She has been ward and theatre sister at the Cheltenham General Hospital, night sister at the Deaconess Hospital, Edinburgh, and assistant matron of the Royal Orthopædic Hospital, Birmingham.

MOUNT VERNON HOSPITAL, NORTHWOOD.—Miss A. C. Knight has been appointed night sister. She was trained at St. Bartholomew's Hospital, and has been night sister at the North Eastern Fever Hospital.

PETERBOROUGH GENERAL INFIRMARY.—Miss M. E. Pewter has been appointed night sister. She was trained at the Royal Hospital, Portsmouth, and has been theatre sister at the Essex County Hospital. She holds the C.M.B. certificate.

NOTTINGHAM GENERAL HOSPITAL.—Miss Alice Low has been appointed home sister. She was trained at the General Hospital, Wolverhampton, and has been sister of the pensioners' ward in the Nottingham General Hospital.

PRINCESS MARY'S HOSPITAL, MARGATE.—Miss Edith Anderson and Miss Lily Steirn, staff nurses, have been appointed sisters. The former was trained at the Manchester Royal Infirmary and Leeds City Hospital, and has been staff nurse at the Horton War Hospital. The latter was trained at King Edward VII. Hospital, Windsor, and the Hospital for Hip Disease, Sevenoaks, and has been staff nurse at the Paddington Military Hospital.

STAFFORDSHIRE GENERAL INFIRMARY.—Miss M. G. Poole has been appointed matron and superintendent of nursing. She was trained at the Wolverhampton and South Staffordshire Hospital, and was successively sister-in-charge of the isolation block, the men's medical ward, and the operating theatre. She was called up for service in the Territorial Force at the outbreak of war, and served at the 5th Northern General Hospital as sister-in-charge of the operating theatre. She served last year at the Norfolk and Norwich Hospital, and there added to her experience in housekeeping.

THE VICTORIA HOSPITAL, KEIGHLEY.—Miss Bessie Liddington has been appointed theatre sister, and Miss Edith Leneghan night sister. They were both trained at the hospital, and Miss Liddington follows her sister as theatre sister.

VICTORIA HOSPITAL, BURNLEY.—Miss Margaret Alexander has been appointed matron. She was trained at the General Hospital, Birmingham, where she has been assistant matron. During the war she served for four and a-half years in France with great distinction, being mentioned three times in despatches, awarded the Royal Red Cross, 2nd Class, in June 1916, promoted to the 1st Class in January 1919, and later awarded a bar. By the French Government she was awarded the Medaille des Epidemies.

UNION HOSPITAL, DARTFORD.—Mrs. Daisy Manley has been appointed superintendent nurse. She was trained at Warrington Union Infirmary, and has been superintendent nurse at Wolstanton and Burslem Union, and at the Isle of Thanet Union.

June 25, 1921] [Price Twopence

### The Hospital

**RATES OF SUBSCRIPTION** (post free, payable in advance).
Three months, 3/3; six months, 6/6; twelve months, 13/-. (Should the cost of printing and paper as well as increased postage, necessitate alteration of these rates, the period paid for will be reduced proportionately on unexpired subscriptions.) THE HOSPITAL "Index" to Volume LXIX., October 1920 to March 1921, is now ready, price 1/1 per copy, post free. Address The Manager, THE HOSPITAL, "The Hospital" Building, 28/29 Southampton Street, Strand, London, W.C. 2.

